# The bionorte network for biodiversity and biotechnology RESEARCH in the brazilian amazon states

**DOI:** 10.1186/1753-6561-8-S4-O46

**Published:** 2014-10-01

**Authors:** Spartaco Filho, Cristina Bosco, Patricia Albuquerque

**Affiliations:** 1 UFAM, BIONORTE, Manaus, Amazonas, Brazil; 2 CGEC/SEPED/MCTI, BIONORTE, Manaus, Amazonas, Brazil; 3 UFMA, BIONORTE, Manaus, Amazonas, Brazil

## 

Created in 04/12/2008 by the Ministry of Science, Technology and Innovation (MCTI) in collaboration with the State Secretaries for Science and Technology (SECTs) from the nine States of Legal Amazon by MCTI decree n^o^. 901. The BIONORTE consortium has the objective of integrate expertise for research, development, innovation and also to graduate personnel at doctorate level. The initiative is focusing in biodiversity and biotechnology and aims to generate knowledge, process and products which can contribute to the sustainable development of the Brazilian Amazon region.

The BIONORTE is lined by MCTI, and managed by a Director Council, composed by representatives of MCTI, MDIC, MI, CONSECTI, CONFAP, CNPq, FINEP, CAPES, FOPROP, Institutions of Research and Education, Entrepreneurs and Companies. BIONORTE is instrumented by a Scientific Committee, composed by representatives from the academic community from all the member States and by their local Scientific Committees and managed by a Executive Coordination in a MCTI division (CGEC/SEPED).

So far the BIONORTE developed two main initiatives that resulted in:

## Graduate program of biodiversity and biotechnology (PPG-BIONORTE)

This graduate program was approved by CAPES in October of 2011 with concept 4 (in a scale of seven) and initiated its classes in March of 2012. By now the BIONORTE program has one hundred fifty professors from eighteen main Amazonian institutions. It has thirty eight courses (five as obligatory and thirty three non obligatory) working in two major areas with three research lines as followed:

Major area: Biodiversity and Conservation

-*research line 1: Knowledge of Amazon Biodiversity*

-research line 2: Sustainable Use and Conservation of Amazon

Major area: Biotechnology

-*research area 1: Bioprospection; Bioprocesses and Bioproducts Development*

At the moment, by the end of its second year, the BIONORTE counts with one hundred eight two graduate students. For 2014 more hundred seats are available and the selection process for new doctorate grad students is in line.

## Integrated research projects

Even before PPG-BIONORTE was created, the Amazonian network was establishing its activities. A public call for projects was launched by CNPq (MCT/CNPq/FNDCT/CT-AMAZÔNIA/BIONORTE - Nº 66/2009) for integrated R&D projects in a framework requirement: the candidates should be from Amazonian States and the subjects need to cover the fields of biodiversity, conservation and biotech. The total number of subscriptions reached seventy projects from which, twenty four were recommended and twenty were hired. The financial resource was provided by the Sectorial Funding from MCTI, SECTs and State Research Funding Agencies (FAPs) from the Amazonian member States. The hired projects have been granted for equipments, consumables and scholarships. Due to its successful results, Brazilian government launched the second version of this public projects call, also through CNPq with the Sectorial Funding (MCTI/CNPq/FNDCT - Ação Transversal - Nº 79/2013), contemplating once again the BIONORTE network. This call was opened until November 4th and there is chance for hiring new R&D projects yet this year.

Along with all these results and activities we can state the BIONORTE initiative is in the right track for its successful consolidation! If you want to know more about us, please go to the BIONORTE site at http://www.bionorte.org.br.

